# Pharmacy workforce: a systematic review of key drivers of pharmacists’ satisfaction and retention

**DOI:** 10.1080/20523211.2025.2470848

**Published:** 2025-02-28

**Authors:** Muna Barakat, Mohammed Sallam

**Affiliations:** a Department of Clinical Pharmacy and Therapeutics, Faculty of Pharmacy, Applied Science Private University, Amman, Jordan; b Department of Pharmacy, Mediclinic Parkview Hospital, Mediclinic Middle East, Dubai, United Arab Emirates; c Department of Management, Mediclinic Parkview Hospital, Mediclinic Middle East, Dubai, United Arab Emirates; d Department of Management, School of Business, International American University, Los Angeles, CA USA; e College of Medicine, Mohammed Bin Rashid University of Medicine and Health Sciences (MBRU), Dubai, United Arab Emirates

**Keywords:** Pharmacy, job satisfaction, workforce retention, leadership, motivation and recognition, staff engagement, training and support

## Abstract

**Background:**

Pharmacy workforces are central to healthcare systems, yet the profession faces challenges in job satisfaction and retention due to evolving roles, workload pressures, and other issues. Understanding workforce stability is crucial for optimising pharmacy services.

**Objective:**

This systematic review aimed to identify and analyze the critical factors impacting pharmacy staff job satisfaction and retention, providing actionable insights to improve workforce stability and long-term engagement in the profession.

**Methods:**

A comprehensive search was conducted following the Preferred Reporting Items for Systematic Reviews and Meta-Analyses (PRISMA), covering broad academic databases including EMBASE, Web of Science, PubMed, International Pharmaceutical Abstracts, and the supplementary use of Google Scholar for studies published between 2019 and 2024. The quality of the included articles was evaluated, revealing a generally low to moderate risk of bias.

**Results:**

The review synthesised findings from 81 studies and extracted ten relevant themes. Countries including the United States, Saudi Arabia, Nigeria, Pakistan, and Southeast Asia countries contributed most frequently, highlighting regional research diversity. Key factors influencing job satisfaction included burnout, stress, and workload (24%); work conditions and roles (22%); professional development (14%); earnings and benefits (10%); and leadership support (9%).

**Conclusion:**

With a global perspective that travels across 36 countries in five continents, this study is the latest in-depth analysis of factors influencing job satisfaction in the pharmacy workforce. This review emphasises the need for policy reforms and further research on workplace conditions in different locations. It provides insights for policymakers and healthcare leaders to enhance the pharmacy workforce's strategic support and engagement initiatives.

## Introduction

Job satisfaction is the extent to which individuals feel positive or negative about their jobs and the degree of satisfaction within their work environment (Al-Muallem & Al-Surimi, 2019). It encompasses an emotional evaluation of their job circumstances (Chua et al., 2013). Pharmacists remain on the frontline of public health around the globe, and their performance directly impacts patients’ safety (Sharma et al., 2024). The leadership at any healthcare facility acknowledges the pharmacist's role in advancing medication safety practices (Sallam & Hamdan, 2023). This acknowledgment highlights the significance of cultivating a supportive workplace that improves job satisfaction for pharmacists and promotes their active involvement in advancing optimal patient outcomes (Liu & White, 2011).

### Background

The pharmacy profession is rapidly expanding, with the growth of specialisation and research activities (Ignoffo et al., 2022; Jones et al., 2024). Pharmaceutical experts often engage in conversations about job fulfilment due to the constant changes in the field (Alshammari et al., 2021). Pharmacy staff job satisfaction is a complex and evolving concept closely tied to opportunities for professional development and role advancement (Slimane, 2017). It also positively relates to motivation, performance, productivity, organisational commitment, and patient safety culture (Al-Surimi et al., 2022; Muin et al., 2019). Acknowledging pharmacists’ rights significantly impacts their satisfaction (Abujarad Alhuwitat et al., 2016). Effective communication and interpersonal relationships are essential in determining the level of career satisfaction within pharmacy (Alomi, Bahadig, Qaism, et al., 2019).

Recruiting and retaining pharmacists remains a global challenge, requiring meaningful strategies to address workforce shortages and ensure continuity of care (Terry et al., 2021).

Identifying the factors affecting job satisfaction and organisational commitment is important to decrease pharmacists’ turnover, increase productivity, and ensure the long-term sustainability of global pharmacy practices (Meilianti et al., 2022; Thin et al., 2022). Additionally, understanding job satisfaction will allow employers to address their employees’ requirements, reduce turnover, and enhance the workplace atmosphere (Thin et al., 2022). The levels of job satisfaction among pharmacists practicing in various settings vary (Khalidi & Wazaify, 2013). Al-Jumaili, Sherbeny, et al. (2022) researched 18 Arab countries and discovered notable differences in job satisfaction among pharmacists in various sectors. Those working in pharmaceutical marketing, academia, and the pharmaceutical industry reported the highest satisfaction levels due to better income and prospects for advancement.

Conversely, community pharmacists and those in administrative roles in the government reported lower satisfaction, linked to workload pressures and inadequate organisational support. The study also revealed that pharmacists with postgraduate degrees and male pharmacists experienced higher satisfaction levels, with professional commitments and organisational factors playing a vital role. Stress and workload can significantly impact job satisfaction as distinct influencing factors (Schommer et al., 2020; Tentama et al., 2019). Slimane (2017) stressed the importance of addressing discontent resulting from colleagues’ hygiene factors, salary, and job security while concurrently increasing contentment through motivator factors such as promotion, recognition, and the inherent nature of the work. Summerlin et al. (2024) surveyed pharmacy technicians and found that barriers such as cost, time, and perceived value limit participation in continuing education. They recommended enhanced guidance, mentorship, and tailored training programmes to improve job preparedness. Desselle (2016) identified key factors influencing pharmacy technician work-life, including career impetus, job responsibilities, quality of work-life, and equitable partnerships, highlighting the importance of these elements in enhancing job satisfaction and organisational commitment. A study by Iorga et al. (2017) on hospital pharmacists in Romania highlighted dissatisfaction with budgets, working hours, and legislation, emphasising the need for policy reforms and improved management strategies to enhance job satisfaction. Aspden et al. (2021) identified dissatisfaction with the professional environment, remuneration, and limited career pathways as key factors influencing pharmacists to leave the profession. Cherezova and Khalimanenko (2021) emphasised combining tangible and intangible incentives to enhance pharmacy staff motivation, improve job satisfaction, and support professional development. Liu and White (2011) identified intrinsic aspects of job satisfaction, such as ability utilisation and recognition, as key factors influencing the job satisfaction of hospital pharmacy staff, emphasising the need for management to focus on providing meaningful work and skill development opportunities. Yong et al. (2020) used role theory to identify factors causing stress and strain for community pharmacists, categorising 41 factors into interpersonal, social, individual, and extra-role attributes. Al-Jumaili et al. (2023) identified role undervaluation, low pay, and high workloads as key contributors to pharmacist dissatisfaction in the Arab world, urging action to improve conditions and patient care. Khalifa and Ben Slimane (2016) highlighted that financial rewards and job-related motivators, such as recognition, feedback, and autonomy, significantly influence pharmacists’ job satisfaction and retention in Saudi hospitals. Creative management techniques are essential for improving the quality of hospital pharmacy operations and increasing staff satisfaction (Alkeelani et al., 2025; Sallam, 2024a; Sallam, Allam, et al., 2024).

### Objectives

It is crucial to consider both internal and external satisfaction factors to retain pharmacists and the need for action to achieve resilience and enhance the quality of pharmacy services (Schommer et al., 2020; She, 2022). This review aimed to identify, gather, and analyze key factors influencing job satisfaction and retention among pharmacy staff and to understand how these drivers contribute to workforce stability within the pharmacy profession. This review aimed to synthesise reliable information and provide comprehensive insights into the elements that support or hinder job satisfaction and retention, ultimately informing strategies to strengthen the stability and sustainability of the pharmacy workforce.

## Materials and methods

### Research question

What are the key factors influencing pharmacy staff job satisfaction and retention, and how do these drivers contribute to workforce stability in the pharmacy profession?

### Search strategy

(‘pharmacy workforce' OR ‘pharmacist workforce' OR ‘pharmacists') AND (‘job satisfaction' OR ‘work satisfaction' OR ‘career satisfaction' OR ‘professional satisfaction') AND (‘engagement’ OR ‘employee engagement' OR ‘work engagement') AND (‘retention' OR ‘workforce stability’ OR ‘employee retention' OR ‘turnover') AND (‘key drivers' OR ‘factors' OR ‘determinants') NOT (‘trainees')

### Information sources

The databases for this systematic review included EMBASE., Web of Science (WoS), PubMed/MEDLINE, International Pharmaceutical Abstracts (IPA), and an additional search engine, Google Scholar, which was utilised to ensure a comprehensive search.

### Inclusion and exclusion criteria

For inclusion, the review considered peer-reviewed empirical research articles that provided data on job satisfaction, retention factors, or workforce stability among pharmacy professionals. Studies had to be published in English and include quantitative, qualitative, or mixed-methods research designs that explored relevant themes within various pharmacy settings, such as community, hospital, or academic institutions. The geographical range was worldwide, with no limitations for applicable research conducted between January 2019 and October 2024.

Exclusion criteria included non-empirical articles, opinion pieces, editorials, conference abstracts, and studies not specific to pharmacy staff or that focused on unrelated job sectors. Studies published in languages other than English or lacking primary data relevant to satisfaction and retention were also excluded. Additionally, studies were excluded if they had insufficient methodological rigour, such as unclear study designs, small or non-representative sample sizes, or lack of statistical analysis. Articles focused solely on pharmacy students or trainees without addressing workforce satisfaction or retention were removed. Studies were also excluded if they failed to report key variables, provided incomplete or ambiguous findings, or lacked transparency in methodology, making replication and interpretation challenging. Furthermore, duplicate records of grey literature with unverifiable data were not considered. A structured record of study exclusions was maintained to enhance methodological transparency and ensure that the final selection represents the most reliable evidence on pharmacy workforce satisfaction and retention.

### Review and selection process, data extraction, and synthesis

The selection process for this review followed the structured PRISMA flow diagram, beginning with collecting records from various databases and sources (Page et al., 2021). EndNote 20.5, a reference management tool, was utilised for the organisation and evaluation of titles and abstracts, and it also aided in detecting duplicate entries.

Two independent field expert reviewers evaluated abstracts for the included articles to determine the relevance of each article. Disagreements were resolved through discussion. The potentially relevant articles then underwent a full-text review by the author for final inclusion, with clear documentation provided for any excluded articles.

In parallel, data from the included studies were systematically extracted using an Excel sheet, capturing essential information such as study design, sample size, geographic region, key variables, main findings, and conclusions related to pharmacy staff job satisfaction and retention. The data were then narratively synthesised, summarising the main findings and identifying trends regarding the critical factors influencing job satisfaction and retention across different settings. This approach ensured a transparent and comprehensive analysis, providing a detailed understanding of the various determinants impacting workforce stability and highlighting prevalent gaps in the existing literature, which can guide future research directions.

### Quality assessment

The research articles included in the study were analyzed using the ROBINS-I (Risk of Bias in Non-randomized Studies of Interventions) and ROB 2 (Risk of Bias 2) tools, respectively (Flemyng et al., 2023; McGuinness & Higgins, 2020; Sterne et al., 2016).

Cohort and Case–Control Studies were evaluated with the Newcastle-Ottawa Scale (NOS), which assesses the selection of study groups, the comparability of the groups, and the ascertainment of the outcome or exposure (Lo et al., 2014). These tailored tools and rigorous assessment ensured a systematic and standardised evaluation of the risk of bias and overall quality, which is crucial for accurately interpreting the systematic review results and including reliable and high-quality articles, strengthening research findings.

### Data analysis

The gathered data were compiled in a summary table, and subsequently, manual content analysis was employed for the classification, mapping, and graphics for the findings using Microsoft Excel 2021 (Godino, 2023).

## Results

**Figure 1.**
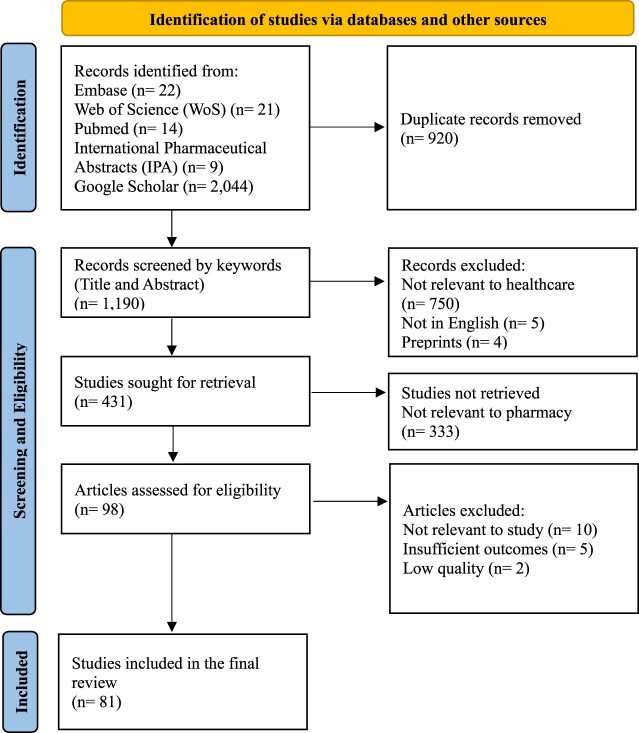
PRISMA (Preferred Reporting Items for Systematic Reviews and Meta-Analyses) flow diagram for the selection process in the study.

The initial database search yielded 2,110 records. After a comprehensive screening process, 81 studies met the inclusion criteria and were deemed eligible for the review. The detailed record selection process is illustrated in the PRISMA flowchart Figure 1.

### Overview of included studies

Table 1 efficiently arranges and displays the included studies for easy access and comparison. The studies examine factors that impact job satisfaction, retention, and professional fulfilment in various pharmacy settings worldwide. These studies cover multiple countries, research methods, and work environments, focusing on wages, workload, recognition, leadership, and workplace atmosphere. The results show that while pharmacists often express dissatisfaction with their pay and opportunities for career advancement, autonomy, professional growth, and supportive leadership play a significant role in enhancing job satisfaction. Proposed solutions commonly include the implementation of structured career paths, improved organisational support, and addressing issues like excessive workloads and lack of recognition.

**Table 1.** Features and results of the included studies.

Author(s)	Country	Journal Name	Year	Study Details	Study Objective	Outcome Measured	Results/Findings
Asghar et al. (2024)	Pakistan	Research in Social and Administrative Pharmacy	2024	Qualitative study using the COM-B model with in-depth interviews among community pharmacists in southern Punjab, Pakistan.	To explore factors influencing community pharmacist retention.	Factors affecting retention and motivators among community pharmacists.	Retention was impacted by training, social acceptance, and consistent salaries, while motivators included educational background, job opportunities, patient interactions, and flexible schedules. Recommendations for regulatory improvements were made.
Zbyrak et al. (2024)	United States	Journal of the American Pharmacists Association	2024	Cross-sectional study using data from the 2018 Virginia Pharmacy Workforce Survey to assess job satisfaction among board-certified pharmacists.	To identify factors associated with job satisfaction among board-certified pharmacists in Virginia.	Job satisfaction levels are influenced by income, hours worked, work setting, and benefits like paid sick leave.	High job satisfaction (93.6%) was reported, with significant associations for higher satisfaction among those in academia and health systems, those earning above $150,000, working 30–49 hours, and receiving paid sick leave.
Gulbis et al. (2024)	United States	Journal of the American College of Clinical Pharmacy	2024	A multi-step approach is used to assess oncology pharmacists’ job satisfaction and retention factors through surveys and workshops.	To identify factors impacting job satisfaction and retention among oncology pharmacists.	Factors related to practice model, professional development, burnout/well-being, and metrics.	Practice model and well-being support were the highest dissatisfiers. Recommendations included redefining practice models, enhancing professional development opportunities, and creating supportive well-being measures.
Thames et al. (2024)	United States	American Journal of Health-System Pharmacy	2024	Implementation of a pharmacy technician career ladder and training programme at U.F. Health Shands Hospital to improve retention and job satisfaction.	To evaluate the impact of a structured career ladder and training programme on pharmacy technicians’ recruitment, retention, and job satisfaction.	Retention rates, job satisfaction, and turnover.	The career ladder and training programme resulted in a 51% reduction in turnover, improved job satisfaction (as measured by employee engagement scores), and decreased overtime hours by 59%, highlighting benefits in recruitment and retention efforts.
Cherecheș et al. (2024)	Romania	Pharmacy	2024	Netnographic analysis of social media discussions among pharmacists on Reddit and Facebook focusing on professional satisfaction and challenges.	To identify key factors influencing professional satisfaction among pharmacists.	Job satisfaction factors including workload, job autonomy, and work-life balance.	Findings indicated dissatisfaction with salaries, lack of professional recognition, and high workload as primary issues affecting satisfaction; suggested improvements included policy reforms and enhanced organisational support.
Shuleta-Qehaja and Kelmendi (2024)	Kosovo	Multidisciplinary Science Journal	2024	A cross-sectional survey was conducted to assess job satisfaction among pharmacists in Kosovo using a web-based questionnaire.	To evaluate job satisfaction levels and influencing factors among pharmacists in Kosovo.	Job satisfaction, factors such as pay, advancement, and workload.	Findings showed moderate satisfaction (59.3%) with significant dissatisfaction due to low pay (46.2%), limited advancement opportunities (42.3%), and high workload (37.4%), recommending improvements in these areas.⁣
Kusumah Wardani et al. (2024)	Indonesia	Journal of Medicine and Pharmacy Faculty	2024	Cross-sectional study with 145 community pharmacists in West Java using the Warr-Cook-Wall instrument.	To determine job satisfaction among community pharmacists and identify influencing factors.	Job satisfaction, intrinsic and extrinsic factors	Pharmacists reported higher satisfaction with intrinsic factors (mean 3.04) than extrinsic factors (mean 3.0), with lower satisfaction in recognition and income; significant factors included working hours and pharmacy ownership type.⁣
Ulutaş Deni̇z et al. (2024)	Turkey	Journal of Research in Pharmacy	2024	A cross-sectional study of 82 pharmacists in Erzurum, Turkey, assessed emotional intelligence's impact on job satisfaction.	To explore the influence of emotional intelligence on pharmacists’ job satisfaction.	Job satisfaction and emotional intelligence	A moderate positive relationship was found between emotional intelligence and job satisfaction, indicating that higher emotional intelligence can enhance job satisfaction, suggesting benefits for pharmacists’ work life and stress management.⁣
Kassaw et al. (2024)	Ethiopia	Health Science Reports	2024	A cross-sectional survey of 217 pharmacy professionals in public and private sectors in Gondar town	To evaluate and compare job satisfaction among pharmacy professionals	Job satisfaction levels, factors associated with satisfaction	47.5% were satisfied; private sector workers had higher satisfaction. Key factors included salary and job location.
Abdullahi et al. (2023)	Nigeria	Exploratory Research in Clinical and Social Pharmacy	2023	A cross-sectional study was conducted to analyze job satisfaction among pharmacists in public health facilities in Nigeria using an online questionnaire.	To examine job satisfaction levels and identify factors influencing job satisfaction among pharmacists in Nigeria's public health facilities.	Job satisfaction focuses on facility conditions, coworker relations, remuneration, and turnover intention.	The study found low overall job satisfaction (53.1%) with significant dissatisfaction in remuneration and facility conditions. Younger pharmacists reported higher satisfaction with coworker relations, while older pharmacists showed declining satisfaction over time.
Bondi et al. (2023)	United States	Journal of the American College of Clinical Pharmacy	2023	A survey of 571 clinical pharmacists was conducted on rewards, recognition, and advancement practices in their institutions.	Examine the current state and preferences for rewards and recognition among clinical pharmacists.	Financial, personal, and professional rewards and recognition preferences	Financial incentives, autonomy, and work-life balance were the most desired rewards; however, less than half of participants reported satisfaction with current recognition and advancement opportunities⁣
Ooi et al. (2023)	Malaysia	Pharmacy	2023	A cross-sectional study used a self-administered questionnaire among community pharmacists in Kuala Lumpur and Selangor, Malaysia.	To assess factors affecting job retention among community pharmacists in Malaysia.	Job retention, work-life balance, value and trust, and career growth factors.	Job retention was strongly linked to value and trust, work-life balance, and career development, with gender, age, and experience also predicting retention rates. Recommendations included boosting workplace support and career advancement opportunities.⁣
Islam and Naqvi (2023)	Saudi Arabia	PLOS ONE	2023	A cross-sectional survey was conducted among community pharmacists in three cities in Saudi Arabia.	To evaluate job satisfaction determinants among pharmacists in Saudi pharmacy settings.	Communication, fringe benefits, and nature of work's impact on job satisfaction.	Communication, fringe benefits, and nature of work had the highest contributions to job satisfaction, with overall satisfaction scores low, emphasising the need for improved workplace support and engagement strategies.⁣
Tran et al. (2023)	Vietnam	PLOS ONE	2023	A cross-sectional study was conducted with 235 hospital pharmacists across 17 Mekong Delta, Vietnam healthcare facilities.	To assess job satisfaction levels and the factors influencing satisfaction among hospital pharmacists.	Factors impacting job satisfaction, including working conditions, leadership styles, and benefits.	Most hospital pharmacists reported high satisfaction (80.4%). Key factors contributing to satisfaction included favourable working conditions, effective leadership, and adequate benefits, with a stronger impact on private settings than public.⁣
Alotaibi et al. (2023)	Saudi Arabia	Cureus	2023	A cross-sectional study involving 2,002 health practitioners in Saudi Arabia assessed the determinants of job satisfaction.	To explore job satisfaction determinants and provide strategic recommendations for health workforce planning in Saudi Arabia.	Job satisfaction factors include gender, age, marital status, nationality, specialty, type of facility, and income.	Significant determinants included gender, with males generally more satisfied; age, with older practitioners more satisfied; and type of facility, with Armed Forces facility practitioners most satisfied. Key recommendations emphasised policy adjustments to improve satisfaction and retention.
Lam et al. (2023)	New Zealand	Pharmacy	2023	Cross-sectional online survey among 694 pharmacists in New Zealand, with comparisons to Canadian data.	To evaluate job satisfaction, work conditions, and psychological distress among pharmacists in N.Z. and compare to Canadian findings.	Job satisfaction, working conditions, psychological distress.	N.Z. pharmacists reported suboptimal working conditions, with many contemplating career changes due to stress; however, they perceived slightly better conditions than Canadian pharmacists. Psychological distress levels remained high.⁣
Lynch and O'Leary (2023)	Ireland	Exploratory Research in Clinical and Social Pharmacy	2023	A qualitative study involving 23 pharmacists to explore factors influencing retention in community pharmacies in Ireland.	To understand the factors affecting retention in the community pharmacy workforce.	Working conditions, career fulfilment, regulatory burden	Identified key retention challenges, including heavy workload, insufficient breaks, limited career progression, regulatory pressures, and lack of professional recognition, contributing to pharmacists’ decisions to leave or stay⁣
Fadare et al. (2023)	United States	Exploratory Research in Clinical and Social Pharmacy	2023	Applied relative importance analysis to assess job satisfaction determinants among community pharmacists.	To identify the key determinants of job satisfaction for community pharmacists using relative importance analysis.	Job satisfaction determinants include workplace discrimination, patient-care services, and compensation.	Workplace discrimination had the highest impact on job satisfaction, while compensation was the least influential; advanced dispensing activities correlated with lower job satisfaction.
Lama et al. (2023)	Lebanon	Journal of Pharmaceutical Policy and Practice	2023	A cross-sectional study with 415 Lebanese pharmacists and fifth-year pharmacy students assessed management/leadership competencies and work satisfaction.	To evaluate management/leadership skills and work satisfaction and establish their association among Lebanese pharmacists.	Management/leadership competencies, work satisfaction	A significant correlation was found between management/leadership skills and work satisfaction. Factors like marital status and education level also impacted satisfaction, emphasising the need to enhance pharmacists’ leadership training.
Abatur et al. (2023)	Nigeria	World Journal of Biology Pharmacy and Health Sciences	2023	A cross-sectional study with 30 pharmacists in a tertiary hospital in Nigeria assessed job satisfaction levels.	To assess job satisfaction and identify factors affecting it among pharmacists in a clinical setting.	Job satisfaction, demographic factors, workplace culture.	Findings indicated low job satisfaction (33.3%), with dissatisfaction linked to hospital culture, work environment, reward system, and remuneration. Recommendations for improving satisfaction included enhancing workplace conditions and salary.⁣
Alanazi et al. (2023)	Saudi Arabia	Saudi Pharmaceutical Journal	2023	Cross-sectional study assessing employee engagement and satisfaction among pharmacy staff at K.A.M.C.	To evaluate employee engagement levels and satisfaction in pharmaceutical care services at King Abdulaziz Medical City (K.A.M.C.).	Employee engagement, job satisfaction, facility rating	Found moderate to high engagement levels among staff, with significant associations between engagement and variables such as occupation and work experience; facility rating averaged 6.51 out of 10, indicating areas for improvement.⁣
Oamen (2023)	Nigeria	Texila International Journal of Management	2023	A longitudinal survey using latent growth curve modelling assesses job satisfaction among pharmaceutical executives across three-time points (June 2021, July 2022, March 2023).	To evaluate changes in job satisfaction among pharmaceutical executives over time.	Job satisfaction growth trajectory using latent growth curve analysis	There was positive growth in job satisfaction over time, indicating that job satisfaction among pharmaceutical executives increased post-COVID, likely due to improved working conditions and adaptive coping strategies. Initial satisfaction did not predict long-term satisfaction growth.⁣
Aridi et al. (2023)	United States	International Journal of Health Systems and Translational Medicine	2023	A qualitative organisational development study explores job satisfaction factors in pharmacy settings with high turnover, utilising action research and content analysis of performance incentives.	Examine factors affecting pharmacists’ job satisfaction and propose organisational development strategies to enhance retention and job satisfaction.	Job satisfaction, retention rates, impact of leadership and incentives	Identified that performance incentives, supportive leadership, and fostering professional development significantly enhance job satisfaction and reduce turnover. Suggested a model focusing on personalised incentives and positive leader-employee exchanges⁣
Antofie et al. (2023)	Romania	Farmacia	2023	A cross-sectional study of 481 pharmacists across various sectors in Romania explored job satisfaction and learning opportunities.	Examine job satisfaction, lifelong learning, and institutional support among Romanian pharmacists.	Job satisfaction, lifelong learning, institutional support	Found low satisfaction with salary but high satisfaction with colleague relationships; a need for supportive laws and continuous education was highlighted to enhance satisfaction and professional development
Etezad et al. (2023)	Canada	Canadian Pharmacists Journal	2023	A cross-sectional survey of 722 community pharmacy professionals in Canada to assess well-being, turnover intention, and patient safety culture.	To explore community pharmacy professionals’ well-being, turnover intentions, and impact on patient safety.	Mental health, turnover intention, patient safety culture	85% reported worsened mental health post-COVID-19, with mental health significantly predicting turnover intention. Work conditions and professional autonomy were found to affect patient safety, suggesting regulatory oversight to improve well-being and reduce turnover risk.
Alshamrani et al. (2023)	Saudi Arabia	Journal of Business and Management Studies	2023	A cross-sectional survey of 325 employees in Saudi pharmaceutical companies exploring factors affecting employee retention.	To identify key determinants of employee retention in Saudi pharmaceutical companies and examine the impact of various factors.	Employee retention, salary, motivation, leadership, culture	Motivation, job conditions, and leadership significantly affect employee retention, with motivation being the strongest predictor. Recommendations include enhancing motivation and work conditions to improve retention rates.⁣
Schlosser et al. (2023)	United States	American Journal of Health-System Pharmacy	2023	A mixed-methods study analyzes job satisfaction and role diversity among pharmacy technicians in ambulatory care across various states.	To assess job satisfaction, varied roles, and commitment levels among ambulatory care pharmacy technicians.	Job satisfaction, role diversity, organisational commitment	Found high job satisfaction linked to technician autonomy, supportive work schedules, and patient-focused roles despite challenges like pay dissatisfaction and workload variability. Emphasised the importance of addressing dissatisfiers to retain technicians and advance the profession
Renger and Niemuth (2023)	Germany	Journal of Nursing & Midwifery Research	2023	The study focuses on job satisfaction among pharmacists in Germany, utilising the K.A.F.A. questionnaire to assess general and specific aspects of job satisfaction in public and non-public pharmacy settings.	To compare job satisfaction between pharmacists in public pharmacies and other professional fields.	Job satisfaction, motivation, turnover	Job satisfaction is high among current pharmacy personnel, but younger generations are less inclined to join the profession. Key factors such as skill variety, autonomy, feedback, and pay significantly influence job satisfaction. High job satisfaction correlates with reduced turnover.
Wong and Kiing (2023)	Malaysia	Malaysian Journal of Pharmacy	2023	A cross-sectional survey of 42 fully registered pharmacists in Miri Hospital, Malaysia, examined job satisfaction, organisational commitment, and intentions to stay.	To assess job satisfaction, organisational commitment, and factors influencing retention among pharmacists in a Malaysian hospital.	Job satisfaction, organisational commitment, intention to stay	Moderate job satisfaction and organisational commitment were reported. Five key factors affecting job satisfaction were identified: workload, co-workers, management treatment, work schedule, and benefits. The study found a strong correlation between job satisfaction and organisational commitment (r = 0.550, *P* < 0.001). Female respondents were more committed to the organisation. Most pharmacists were strongly inclined to remain in their current role, indicating a stable retention outlook among the sample.
Cox et al. (2023)	Australia	International Journal of Pharmacy Practice	2023	A qualitative study with semi-structured interviews of pharmacists who left general practice roles in Canberra within 12 months of starting.	To explore factors contributing to pharmacists leaving general practice	Reasons for leaving and job satisfaction	The main reasons included a lack of role definition, poor utilisation, professional relationship challenges, and insufficient hours; results suggest a need for clear job roles and better integration strategies.⁣
Sola (2023)	Croatia	FIP Pharmacy Practice Research summer meeting for PhD students	2023	An international cross-sectional survey with a 40-item questionnaire was distributed via EPhEU and Google Forms to 811 pharmacists across 16 European countries between October 2022 and January 2023	Assess the differences among the pharmacy workforce in selected domains and evaluate job satisfaction among pharmacists in European countries. Job satisfaction, workload, perceived value of work, public respect, and recognition by the health system.	Job satisfaction, workload, perceived value of work, public respect, and recognition by the health system.	51.3% were satisfied with their job, but only 17.6% felt their work was valued. 84% believed pharmacists are important to the health system, but only 45% thought they had public respect. Only 21% felt the health system recognised pharmacists’ competencies. Primary determinants of job satisfaction were intrinsic aspects of the job, with issues such as high workload, inadequate salaries, and low respect cited as key challenges.
Elshami et al. (2023)	Qatar	American Journal of Pharmaceutical Education	2023	A mixed-methods study involving 136 survey responses and seven focus groups with Qatar University pharmacy alumni focused on job satisfaction, achievements, and preparedness.	To examine job satisfaction, achievements, and preparedness of pharmacy alumni.	Job satisfaction, achievements, and practice preparedness	Alumni showed a high level of job satisfaction, driven by recognition and collaboration, but dissatisfaction stemmed from limited growth opportunities; preparedness for clinical roles was strong, though non-clinical knowledge areas required improvement⁣
Dar-Odeh et al. (2022)	Jordan	Asia Pacific Journal of Health Management	2022	A cross-sectional study was conducted using an online survey among Jordan healthcare professionals (physicians, dentists, and pharmacists). The study included 294 participants.	To evaluate career satisfaction among healthcare professionals in Jordan and identify factors influencing their satisfaction.	Career satisfaction levels and influencing factors	Approximately 56% of participants were satisfied with their careers. Higher satisfaction was associated with marriage, having children, higher income, and homeownership. The employees of academic and non-governmental organizations showed higher satisfaction.
Radwan et al. (2022)	United States	Exploratory Research in Clinical and Social Pharmacy	2022	A cross-sectional survey of Virginia pharmacists analyzed job satisfaction using the 2018 Virginia Pharmacist Workforce Survey data.	To identify predictors of job satisfaction among Virginia pharmacists.	Job satisfaction is influenced by work setting, income, working hours, gender, and patient care involvement.	High job satisfaction was reported, with greater satisfaction among those working fewer hours, earning higher income, and practicing in health systems, clinics, or academia.
Mukosha et al. (2022)	Zambia	International Journal of Pharmacy Practice	2022	A cross-sectional study of 156 pharmacists in Zambia's public and private sectors to assess job satisfaction levels.	To evaluate job satisfaction levels and identify factors influencing satisfaction among Zambian pharmacists.	Job satisfaction concerning sector (public vs. private), income, and job conditions.	Higher job satisfaction was reported in the private sector; income and job stability were key drivers. Pharmacists in the public sector reported lower satisfaction levels, highlighting the need for improved conditions.⁣
Noha Abd Alkareem Younis (2022)	Jordan	Pharmacognosy Journal	2022	A cross-sectional study on job satisfaction among 236 pharmacy certificate holders in Jordan examined various influencing factors.	To assess job satisfaction levels and related factors among Jordanian pharmacy certificate holders.	Job satisfaction and related sociodemographic and work environment factors.	The majority were dissatisfied, with significant factors being low salaries, limited promotion opportunities, and challenging work conditions. Recommendations for policy revisions and improved H.R. practices were suggested to boost satisfaction.⁣
Al-Omar et al. (2022)	Saudi Arabia	Risk Management and Healthcare Policy	2022	Cross-sectional study examining job motivation and satisfaction among female pharmacists in private sectors across Saudi Arabia.	To assess job motivation and satisfaction levels among female pharmacists in the private sector.	Job motivation factors, job satisfaction, and associations with demographics.	Moderate job satisfaction was found, with motivation driven by skill-learning opportunities, international communication, and recognition. Saudi, full-time, and promotion-expecting pharmacists showed higher satisfaction.
Shoji et al. (2022)	Japan	Pharmacy Practice	2022	A cross-sectional survey comparing job satisfaction and expertise demonstration between pharmacists and dietitians in community pharmacies.	To assess job satisfaction and expertise demonstration levels among pharmacists and dietitians in Japan.	Job satisfaction, demonstration of expertise, and work environment factors.	Pharmacists reported higher scores for expertise demonstration, while dietitians had similar levels of job satisfaction. Due to their dual roles as clerks and nutritionists, dietitians found it challenging to apply their expertise fully.
Mishima et al. (2022)	Japan and England	International Journal of Healthcare Management	2022	Qualitative study using interviews to assess opportunities for community pharmacists in Japan and England to demonstrate expertise and how this relates to job satisfaction.	To identify operations viewed as opportunities to demonstrate expertise and job satisfaction sources for community pharmacists.	Opportunities for expertise, job satisfaction, autonomy, and recognition.	Opportunities for demonstrating expertise were linked to job satisfaction but insufficient for satisfaction without autonomy and respect. English pharmacists reported higher satisfaction with formal consultation roles than their Japanese counterparts.⁣
Horodetska et al. (2022)	Ukraine	ScienceRise: Pharmaceutical Science	2022	An online survey was conducted among 508 pharmaceutical professionals on Facebook between April 3 and November 1, 2021.	Investigate using modern motivational tools, including training and social projects, for pharmaceutical specialists.	Motivational factors, effectiveness of training, participation in social and charitable projects, and evaluation of professional activities.	Professional and psychological motivational training ranked highest among educational projects (75.2%). Social and charitable project participation was a motivational advantage (67.5%). Material forms of incentives (73.2%) and innovative training (55.1%) were widely used. These methods positively influenced job satisfaction and organisational commitment in pharmaceutical settings.
Al-Jumaili, Mohammed, et al. (2022)	Iraq	Brazilian Journal of Pharmaceutical Sciences	2022	A cross-sectional survey assessing job satisfaction among pharmacists in Iraq, covering private and public sectors.	To evaluate work satisfaction, characteristics, and job sector differences among Iraqi pharmacists.	Job satisfaction, income, manager satisfaction, expectation alignment.	Approximately 47% of pharmacists were dissatisfied with their primary workplace. Pharmacists in the private sector reported higher satisfaction with income and management, highlighting a preference for private over public sector roles.⁣
Stavrou et al. (2022)	Cyprus	Pharmacy	2022	A cross-sectional survey assessing job satisfaction and stress levels among pharmacists in Cyprus across public and private sectors.	To evaluate job satisfaction and stress levels in pharmacists, comparing public and private sectors.	Job satisfaction, perceived stress, self-efficacy	Pharmacists in private pharmacies reported higher job satisfaction than public sector pharmacists, while public sector pharmacists showed stronger self-efficacy; stress levels negatively impacted job satisfaction.⁣
Mathevula and Dhliwayo (2022)	South Africa	Academic Journal of Interdisciplinary Studies	2022	Cross-sectional survey on job satisfaction of healthcare professionals (doctors, nurses, pharmacists) in South Africa	To determine the levels of job satisfaction among healthcare professionals in South Africa and identify key factors	Job satisfaction factors (salary, benefits, job security, work progression, work recognition)	Most healthcare professionals were dissatisfied with benefits, salary, and work recognition, while job security was a relatively positive factor. Satisfaction levels varied significantly between professions, with pharmacists and nurses showing the highest dissatisfaction levels. Recommendations for improving work conditions were provided.
Widhiandono et al. (2022)	Indonesia	International Business Education Journal	2022	A cross-sectional study with 160 pharmacists in Banyumas Regency, Indonesia, assessed personality, job satisfaction, organisational commitment, and job performance.	To investigate the mediating effect of organisational commitment on the relationship between personality, job satisfaction, and job performance.	Personality traits, job satisfaction, organisational commitment, job performance	Found significant positive effects of personality traits, job satisfaction, and organisational commitment on pharmacists’ job performance, with organisational commitment partially mediating the effects of personality and job satisfaction on performance
Abdullah Nafea Al Shammari (2022)	Saudi Arabia	Chelonian Conservation and Biology	2022	A cross-sectional survey of 500 pharmacy technicians in hospitals and community pharmacies across Saudi Arabia examining job satisfaction, professional development, and medication safety practices.	To identify the factors influencing job satisfaction, professional development, and medication safety practices among pharmacy technicians in Saudi Arabia.	Job satisfaction, professional development, medication safety practices	Key predictors of job satisfaction included autonomy, interpersonal relationships, and workload. Professional development was influenced by access to continuing education and supportive management. Medication safety practices were linked to the availability of technology and staff training.
Ifeoma et al. (2022)	Nigeria	African Journal of Pharmacy and Pharmacology	2022	Cross-sectional survey on job satisfaction and intention to quit among pharmacists across three states in Southeastern Nigeria.	To assess job satisfaction levels and intention to quit among pharmacists and explore contributing factors.	Job satisfaction, intention to quit, demographic influences	Found high satisfaction rates in teamwork and service quality, while low satisfaction in salary and working conditions; nearly half indicated intention to quit, with higher odds among younger and less experienced pharmacists, suggesting retention challenges in Nigeria⁣
Njoku and Ogidi (2022)	Nigeria	Research Journal of Management Practice	2022	A cross-sectional survey was conducted on 153 pharmacists in Jos, Plateau State, Nigeria, assessing job satisfaction, organisational climate, and turnover intention.	To examine the relationship between extrinsic job satisfaction, organisational climate, and turnover intention among pharmacists.	Extrinsic job satisfaction, organisational climate, turnover intention	Found a significant negative relationship between extrinsic job satisfaction and turnover intention, with organisational climate also negatively affecting turnover intention, suggesting a need for improved extrinsic satisfaction to reduce turnover⁣
Citraningtyas et al. (2022)	Indonesia	International Journal on Recent Trends in Business and Tourism	2022	A cross-sectional study involving 25 pharmacists at Manembo-nembo Bitung Hospital assessed the impact of motivation and work discipline on performance.	To determine the effect of motivation and work discipline on employee performance.	Motivation, work discipline, and performance	Found a significant positive effect of motivation (12.3%) and work discipline (42.8%) on performance, highlighting the importance of these factors in enhancing employee performance in hospital settings
Rao et al. (2022)	United States	Journal of the American College of Clinical Pharmacy	2022	A survey of 607 oncology pharmacists in the U.S. assessed factors in job satisfaction, burnout, attrition, and career preferences.	To identify attrition and retention factors among oncology pharmacists.	Job satisfaction, attrition risk, and influencing factors	High satisfaction (78%) was noted, but 60% reported attrition risk, with key drivers being workload, burnout, lack of work-life balance, and ineffective leadership; enhanced clinical engagement correlated with job satisfaction and reduced attrition, highlighting a need for targeted retention strategies including workload management, leadership support, and recognition of pharmacist contributions.⁣
Nguyen et al. (2022)	Vietnam	PLoS ONE	2022	A cross-sectional study surveyed 351 Vietnamese community pharmacists using the validated V.I.J.S. scale with 34 items across six factors.	To develop and validate an instrument (VIJS) for measuring the job satisfaction of community pharmacists in Vietnam during COVID-19.	Job satisfaction across six factors	The VIJS demonstrated strong reliability and validity, indicating high internal consistency and test-retest reliability across six factors, including work conditions, relationships, and opportunities for advancement, despite pandemic-induced challenges.⁣
Terry et al. (2022)	Australia	Rural and Remote Health	2022	Developed the Pharmacy Community Apgar Questionnaire (PharmCAQ) using a modified Delphi technique with expert focus groups and pilot testing in rural Victoria.	To create a tool for supporting recruitment and retention of rural pharmacists.	Factors influencing recruitment and retention	Identified 50 key factors impacting rural pharmacist recruitment and retention across five categories (geographic, economic, practice scope, practice environment, community support); reliability confirmed with a Cronbach's alpha of 0.852⁣
Jairoun et al. (2022)	United Arab Emirates	Pharmacy Practice	2022	A cross-sectional study was conducted on 543 community pharmacists in the U.A.E. to assess over-the-counter (OTC) counseling practices and job satisfaction.	To evaluate the relationship between OTC counseling and job satisfaction among pharmacists.	Job satisfaction and quality of OTC counseling practices	High job satisfaction was associated with better OTC counseling practices, with experienced pharmacists, those in chain pharmacies, and postgraduates displaying superior counseling; dissatisfaction correlated with poorer practices
Aldaiji et al. (2022)	Saudi Arabia	Healthcare	2022	A cross-sectional survey of 284 pharmacists in various sectors in Saudi Arabia to assess the effect of occupational stress on job satisfaction.	To evaluate the relationship between occupational stress and job satisfaction among Saudi pharmacists.	Job satisfaction and occupational stress levels	Occupational stress negatively affected job satisfaction (β = −0.456), with hospital pharmacists reporting the lowest satisfaction; job satisfaction was positively associated with confirmation of expectations
Iheanacho and Odili (2021)	Nigeria	Tropical Journal of Pharmaceutical Research	2021	A cross-sectional survey assessing job satisfaction among 200 pharmacists in various practice areas in Benin City.	To assess job satisfaction and identify key factors impacting pharmacists in Benin City.	Job satisfaction across community, hospital, academia, and industry pharmacy.	Pharmacists in academia reported the highest satisfaction, while hospital pharmacists were the least satisfied. Salary increase, promotion, and recognition were top satisfaction indicators, with motivational talks and leave being the least significant.
Maher et al. (2021)	Palestine	Al-Quds Journal for Academic Research	2021	A cross-sectional survey assessing job satisfaction and stress among 554 West Bank, Palestine registered pharmacists.	To evaluate job satisfaction and job-related stress levels among pharmacists practicing in Palestine.	Job satisfaction, job-related stress, and influencing factors.	The study found moderate job satisfaction levels (58.5%), with significant stress sources including workload, lack of promotion, and poor physician cooperation. Hospital pharmacists reported lower job stress compared to community pharmacists.⁣
Ibrahim et al. (2021)	Iraq	Pharmacy Practice	2021	Cross-sectional survey assessing job satisfaction among community pharmacists in Baghdad, Iraq.	To determine job satisfaction levels and the impact of various demographic factors among community pharmacists in Baghdad.	Job satisfaction and influencing factors, including gender, age, working hours, and years in practice.	Moderate job satisfaction was reported, with gender and age having significant effects. Younger and part-time pharmacists were less satisfied, while those with more experience and full-time roles showed higher satisfaction.⁣
Berassa et al. (2021)	Ethiopia	Journal of Pharmacy Policy and Practice	2021	Cross-sectional study assessing job satisfaction among 80 pharmacy professionals in Tikur Anbessa Specialised Hospital, Ethiopia.	To determine job satisfaction levels and factors influencing satisfaction among pharmacy professionals.	Job satisfaction is related to the working environment, professional interaction, incentives, and recognition.	Near half of respondents were dissatisfied, with main dissatisfaction factors including high workload, inadequate salary, poor respect from management, and insufficient promotion opportunities. Recommendations include improved working conditions and incentives.
Smolina et al. (2021)	Russia	Russian Open Medical Journal	2021	A cross-sectional survey involving 407 pharmacists in Saratov Oblast, Russia, community pharmacies.	To assess job and salary satisfaction among pharmacists and the factors influencing them.	Job and salary satisfaction, along with factors like age, professional commitment, and frequency of job changes.	77.1% of pharmacists were satisfied with their jobs, and 52.8% were satisfied with their salaries. Job satisfaction was influenced by professional commitment, perception of social importance, and age, with younger pharmacists showing higher satisfaction.⁣
Losier et al. (2021)	Canada	Canadian Journal of Hospital Pharmacy	2021	Mixed-methods study assessing job satisfaction among Canadian hospital pharmacists and the impact of clinical pharmacy KPIs.	To determine job satisfaction levels among Canadian hospital pharmacists and examine the influence of cpKPI activities.	Job satisfaction and time spent on cpKPI activities.	Job satisfaction was generally high, with increased satisfaction associated with time spent on cpKPI activities, especially medication reconciliation. However, satisfaction decreased with time spent on drug therapy problem resolution.⁣
Khaliq et al. (2021)	Pakistan	RADS Journal of Pharmacy and Pharmaceutical Sciences	2021	A cross-sectional survey assessing compensation and job satisfaction among 301 pharmacists in various sectors in Karachi, Pakistan.	To evaluate compensation levels and job satisfaction among pharmacists in Pakistan.	Compensation satisfaction, job satisfaction, sectoral differences.	The study found that 57% of pharmacists were dissatisfied with their salaries, with only 34.55% reporting satisfaction. Private sector pharmacists had higher compensation than government or N.G.O. sectors, highlighting a need for pay scale revisions.
Jegede and Ola-Olorun (2021)	Nigeria	Nigerian Journal of Pharmaceutical Research	2021	A cross-sectional survey involved 91 hospital pharmacists in secondary and tertiary hospitals in Osun State.	To assess job satisfaction levels among hospital pharmacists and factors influencing it.	Job satisfaction, remuneration, advancement opportunities, work environment	Hospital pharmacists were generally satisfied with their roles; however, remuneration, lack of advancement opportunities, and time for personal life were areas needing improvement to boost overall satisfaction.⁣
Carvajal et al. (2021)	United States	Pharmacy	2021	Cross-sectional survey assessing gender differences in job and career satisfaction among U.S. pharmacists.	To evaluate and compare career satisfaction and its determinants among male and female pharmacists in the U.S.	Career satisfaction, job satisfaction by gender	Female pharmacists reported higher levels of career satisfaction than male pharmacists; factors like age, marital status, earnings, and job type influenced satisfaction differently between genders.⁣
Clabaugh et al. (2021)	United States	Journal of the American Pharmacists Association	2021	A mixed-methods study surveyed 1,222 community pharmacists in the U.S. on perceptions of working conditions, fear of discipline, and job satisfaction, with Likert-type responses.	Examine pharmacists’ perceptions of working conditions, safety concerns, and job satisfaction.	Working conditions, fear of discipline, job satisfaction	Pharmacists in chain pharmacies reported more negative perceptions of working conditions and a high fear of discipline when addressing safety concerns; common issues included excessive workload, lack of support for safety policies, and low job satisfaction, especially among those working in high-prescription-volume settings⁣
Nguyen-Thi et al. (2021)	Vietnam	PLoS ONE	2021	A cross-sectional study with 197 clinical pharmacists in Ho Chi Minh City, Vietnam, assessed job satisfaction factors via self-administered surveys.	To examine the job satisfaction of clinical pharmacists and associated factors	Job satisfaction and influencing factors	High job satisfaction (74.1%) among clinical pharmacists, with inter-professional relationships as a strong satisfaction driver, while low satisfaction in income and ward round participation were noted as areas needing improvement
Butt et al. (2021)	Pakistan	Pakistan Journal of Medical Sciences	2021	Cross-sectional analysis of job satisfaction among 200 pharmacists working in pharmaceutical sales and marketing in Pakistan, evaluating demographic influences.	To quantify job satisfaction among pharmacists in sales and marketing and its relationship with demographics.	Job satisfaction scores, demographic correlations	The mean job satisfaction score was 2.51 (SD ± 0.49); dissatisfaction was linked to salary issues, job stress, and inadequate incentives, while positive recognition from doctors correlated with higher satisfaction.
Mattsson and Gustafsson (2020)	Sweden	Pharmacy	2020	Cross-sectional alumni survey assessing job satisfaction among pharmacy graduates from Umeå University, Sweden, between 2015 and 2018	To explore and compare job satisfaction among Swedish pharmacy graduates over time.	Job satisfaction, factors influencing satisfaction, access to C.*P*.D.	High job satisfaction was reported (91.4%), with a significant correlation between satisfaction and access to continuous professional development (C.*P*.D.), but no impact of age, gender, or income on satisfaction levels was found.
Zhao et al. (2020)	China	Medicine	2020	A cross-sectional survey assessing burnout and job satisfaction among 1,394 hospital pharmacists at a national conference in China.	To examine the impact of personality, work characteristics, and environment on burnout and job satisfaction.	Burnout dimensions (emotional exhaustion, depersonalisation) and job satisfaction.	High emotional exhaustion (48.2%) and depersonalisation (87.2%) levels were found, with job demands and control influencing burnout. Personality traits like neuroticism predicted burnout, while job satisfaction was linked to job control and work environment.⁣
Ayele et al. (2020)	Ethiopia	Journal of Pharmaceutical Policy and Practice	2020	A cross-sectional study was conducted with 220 pharmacy professionals in eastern Ethiopian public hospitals, assessing job satisfaction levels and associated factors.	To assess job satisfaction levels and determine predictors of satisfaction among pharmacy professionals.	Job satisfaction, with a focus on demographics, work hours, qualifications, and work units.	Low job satisfaction was reported (32.7%), with significant dissatisfaction linked to younger age, bachelor's degree holders, those working over 40 hours per week, and those in dispensing units. Recommendations included workload reduction and improved working conditions.⁣
Iqbal and Iqbal (2020)	Pakistan	Journal of Pharmaceutical Research International	2020	A cross-sectional study was conducted using a self-administered questionnaire distributed to 172 community pharmacists in Pakistan. Data was analyzed using S.*P*.S.S. with statistical significance at *p* ≤ 0.05.	Determine the level of career satisfaction among community pharmacists (CPs) in Pakistan and analyze factors affecting it.	Career satisfaction among Community Pharmacists	The study found that 73.3% of C.*P*.s were unsatisfied with their career. Factors such as age (significant at *p* = 0.009 for pharmacists aged <34) and experience in independent pharmacies (*p* = 0.044) were identified as impacting career satisfaction. Most pharmacists were dissatisfied with their income, feeling that rewards were insufficient. Additionally, 64% reported a lack of recognition and praise in their field, affecting job satisfaction.
Iqbal et al. (2020)	Pakistan	Journal of Pharmaceutical Research International	2020	A cross-sectional survey of community pharmacists using a self-administered questionnaire, with data analyzed using S.P.S.S.	To assess job and workplace satisfaction among community pharmacists and identify predictors affecting satisfaction levels.	Job satisfaction, workplace satisfaction, predictors of satisfaction	High satisfaction (76.7%) was found among community pharmacists, with chain pharmacy pharmacists reporting higher satisfaction than independent pharmacists. Practice setting was a significant predictor of satisfaction, while salary, age, and gender were not.
Lan et al. (2019)	Taiwan	Journal of Occupational Health	2019	A cross-sectional study involving 101 pharmacists from three teaching hospitals in Taiwan was conducted using a structured questionnaire.	To examine the relationship between organisational climate, job stress, workplace burnout, and retention of pharmacists.	Retention, Job Stress, Workplace Burnout	A positive organisational climate was associated with higher retention. Job stress and burnout negatively impacted retention intentions. High burnout correlated with higher turnover intention
Al-Omar et al. (2019)	Saudi Arabia	Saudi Pharmaceutical Journal	2019	Cross-sectional survey study on the impact of perceived organisational support and resilience on pharmacists’ engagement in Saudi Arabia.	To explore the relationship between perceived organisational support, resilience, and employee engagement among Saudi pharmacists.	Perceived organisational support, resilience, and employee engagement.	Results showed that perceived organisational support was significantly correlated with employee engagement, while resilience did not show a statistically significant relationship. Recommendations included enhancing organisational support to improve engagement.⁣
Alomi, Rph, Alghuraybi, et al. (2019)	Saudi Arabia	Pharmacology, Toxicology and Biomedical Reports	2019	A cross-sectional survey of 242 pharmacists in Saudi Arabia assessing stress factors and job satisfaction.	To explore the impact of stress factors on pharmacist job satisfaction in Saudi Arabia.	Job satisfaction related to pharmacy management policies, stress, and motivation factors.	Pharmacists reported moderate job satisfaction. Key stressors included excessive workload and low salary. Financial rewards and flexibility in work schedules were significant motivators for job satisfaction.⁣
Teong et al. (2019)	Malaysia	Journal of Pharmacy Practice and Research	2019	A cross-sectional study was conducted with 286 community pharmacists in Klang Valley, Malaysia, assessing job satisfaction and stress levels.	To evaluate job satisfaction and stress among community pharmacists in an urban Malaysian area.	Job satisfaction, job stress, and demographic influences	Found moderate job satisfaction (mean score 3.39/5) with highest satisfaction in the work environment and interpersonal relations; stress primarily from patient care responsibilities. Demographics such as ethnicity and workload affected satisfaction and stress⁣
Azmi et al. (2019)	Malaysia	International Journal of Human Resource Studies	2019	Cross-sectional study assessing factors affecting retail pharmacists’ retention in Malaysia, focusing on transformational leadership, job satisfaction, and procedural justice, with continuance commitment as a mediator.	To identify the factors influencing retention among retail pharmacists in Malaysia and examine the mediating role of continuance commitment.	Employee retention, job satisfaction, leadership, procedural justice, commitment	Found that job satisfaction, transformational leadership, and procedural justice significantly influenced retention, with continuance commitment playing a mediating role, highlighting the importance of organiational factors in pharmacist retention⁣
Dung et al. (2019)	Vietnam	The South East Asian Journal of Management	2019	A cross-sectional study with 300 pharmacists from various organizations in Vietnam used S.E.M. to analyze factors influencing organisational commitment.	To investigate the impact of job satisfaction, leadership styles, and demographic variables on organisational commitment among Vietnamese pharmacists.	Organisational commitment, job satisfaction, leadership styles, demographic factors	Found that job satisfaction, leadership styles, and demographic variables significantly influenced pharmacists’ organisational commitment, with marital status, age, and tenure being particularly impactful⁣
Alomi, Bahadig, Shahzad, et al. (2019)	Saudi Arabia	Pharmacology, Toxicology and Biomedical Reports	2019	Cross-sectional survey assessing pharmacy practice factors affecting pharmacy technician job satisfaction across Saudi Arabia.	To explore the impact of various pharmacy practice factors on job satisfaction among pharmacy technicians in Saudi Arabia.	Job satisfaction levels concerning management, structure, informatics, and clinical activities.	Found that pharmacy technician job satisfaction was influenced negatively by inadequate pharmacy informatics, inventory management, and lack of clinical pharmacy roles, emphasising the need for improved services to boost satisfaction
Danganan et al. (2019)	Philippines	Journal of Asian Association of Schools of Pharmacy	2019	A descriptive, correlational study assessing quality of work (QWL) among 292 pharmacists using stratified random sampling in the Philippines.	To evaluate the quality of work of pharmacists in the Philippines, focusing on stress, control, job satisfaction, commitment, and work-home conflict.	Quality of work-life dimensions (stress, job satisfaction, work-home conflict, etc.)	Pharmacists who experienced moderate stress and control in their work environment had positive job satisfaction and professional commitment but reported negative work-home conflict, with significant correlations among stress, control, satisfaction, and commitment dimensions.
Ghayas et al. (2019)	Pakistan	International Journal of Scientific & Engineering Research	2019	A cross-sectional survey with 200 pharmacists from Karachi's industrial and hospital pharmacy sectors analyzed factors influencing job satisfaction.	To evaluate factors contributing to job satisfaction levels among pharmacists.	Job satisfaction levels and key factors	Factor analysis identified eight major components contributing to job satisfaction: learning, job timing, change of duties, use of skills, benefits, and career opportunities. Key factors include employee benefits, respect for females, stress levels, and career advancement opportunities.
Rijaluddin et al. (2019)	Indonesia	Journal of Basic and Clinical Physiology and Pharmacology	2019	A cross-sectional survey with 507 community pharmacists in East Java examined various barriers impacting job satisfaction.	To explore the barriers affecting job satisfaction among community pharmacists in East Java, Indonesia.	Job satisfaction, barriers (e.g. lack of recognition, workload)	Nine dominant barriers were identified, including lack of time for patient interaction, insufficient recognition, and poor infrastructure, all contributing significantly to lower job satisfaction. Emphasised the need for systemic changes to improve job satisfaction
Gu and Itoh (2019)	Japan and China	International Journal of Health Planning and Management	2019	A comparative study using cross-sectional surveys among healthcare employees in Japan and China, including 474 Japanese and 429 Chinese participants.	To compare job satisfaction levels and crucial predictors of job satisfaction among healthcare employees in Japan and China.	Job satisfaction factors, levels, and predictors	The study identified shared satisfaction factors, including growth, workload, and reputation. Key differences included the significance of financial rewards and patient relationships in China. Japanese physicians were more satisfied, while Chinese nurses reported higher satisfaction.

Furthermore, there were differences between regions, with private-sector pharmacists reporting higher satisfaction due to better pay and resources than their public-sector peers. The unique challenges faced by rural pharmacists, community pharmacists, and those in hospital-specialised fields like oncology underscore the need for tailored strategies to enhance job satisfaction and retention. These findings emphasise the importance of addressing systemic issues in global pharmacy practice, promoting better working conditions, and supporting long-term workforce stability.

### Risk of bias in included studies

The methodological rigour of the included studies was evaluated using standardised assessment tools specific to the study design (McGuinness & Higgins, 2020; Sterne et al., 2016). While the systematic review is based on largely reliable studies, some caution is warranted in interpreting findings, particularly in areas where methodological inconsistencies or incomplete data reporting were identified.

**Figure 2.**
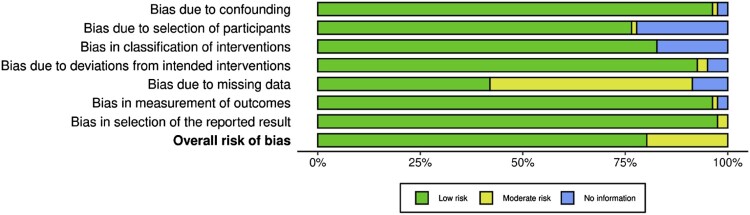
Evaluation of publication quality through a risk-of-bias assessment.

The overall risk of bias assessment revealed that most studies had a low risk across various domains, including participant selection, intervention classification, and outcome measurement. However, moderate risk was noted in handling missing data and deviations from intended interventions. A few studies had areas lacking information, highlighting the need for more transparent reporting in future research. The evaluation of bias risk in the included studies is presented in the provided bar chart in Figure 2.

### Annual trend of published studies from 2019 to 2024

**Figure 3.**
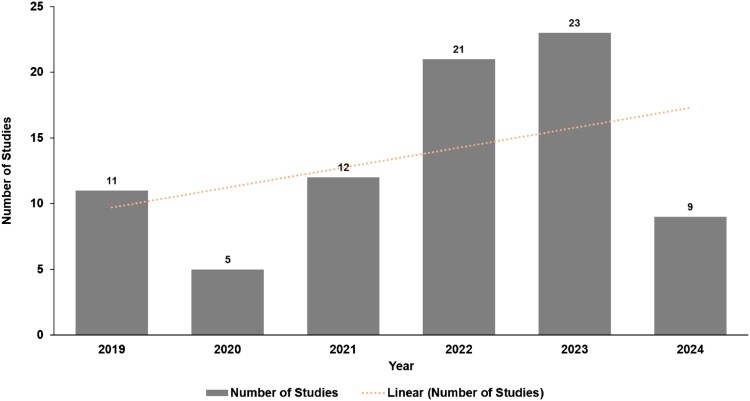
Annual trend of published studies from 2019 to 2024.

The yearly pattern of released research papers from 2019 to 2024 (Figure 3) displays a fluctuating but generally ascending trend. The quantity of papers rose from 11 in 2019 to a peak of 23 in 2023, with a slight decrease to 5 papers in 2020. A steady increase is evident from 2020 to 2023, signifying a growing interest in research in this area. However, the number of papers dropped to 9 in 2024, indicating additional investigation is necessary.

### Global geographical distribution of studies

**Figure 4.**
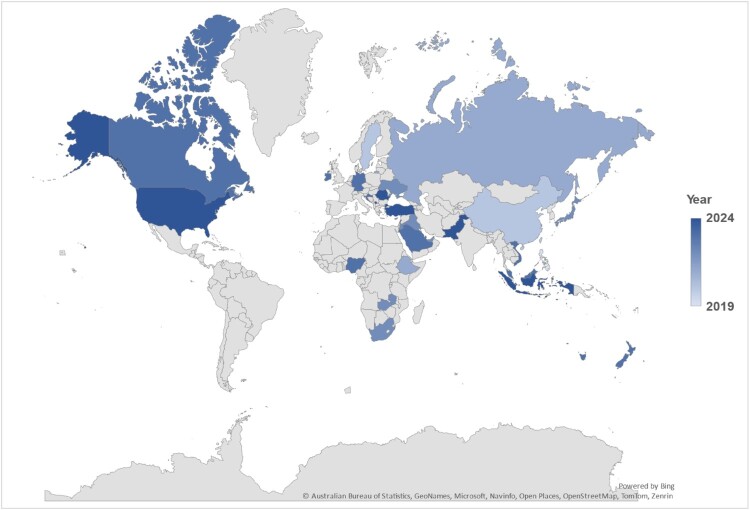
Global geographical distribution of the included studies 2019–2024.

Figure 4 map visually represents the global distribution and publication timeline of studies on pharmacy job satisfaction from 2019 to 2024. Countries like the United States, Saudi Arabia, Nigeria, Pakistan, and Southeast Asia (Malaysia, Vietnam, and Indonesia) exhibit the most frequent contributions, highlighting their active role in pharmacy workforce research. The gradient shading reflects the publication year, with darker shades indicating more recent studies. This map highlights the geographic diversity in addressing pharmacy workforce issues, showcasing significant contributions from developed and developing nations.

**Figure 5.**
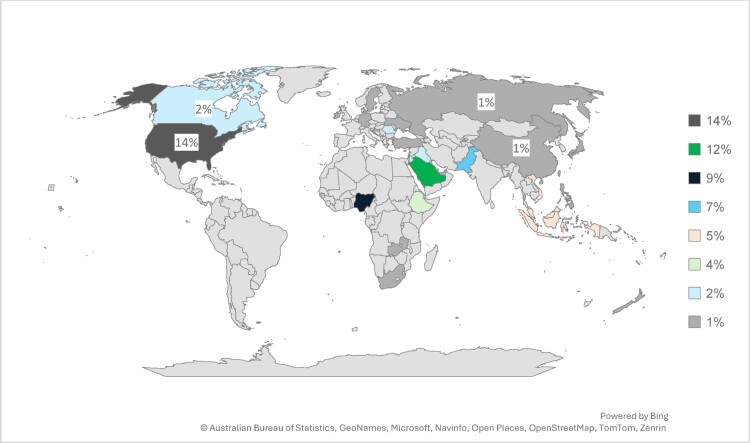
Percentage of global geographical distribution of studies.

The data visualisation in Figure 5 displays the worldwide percentage involvement in pharmacy job satisfaction research, with the United States leading at 14%, followed by Saudi Arabia at 12%, Nigeria at 9%, and Pakistan at 7%. Indonesia, Malaysia, and Vietnam each account for 5%, while Ethiopia contributes 4%. Additionally, Australia, Canada, Iraq, and Romania make smaller but significant contributions at 2%, highlighting the diverse global participation in studying job satisfaction patterns in the pharmacy field.

### Key drivers of pharmacy workforce satisfaction and retention: a Pareto perspective

**Figure 6.**
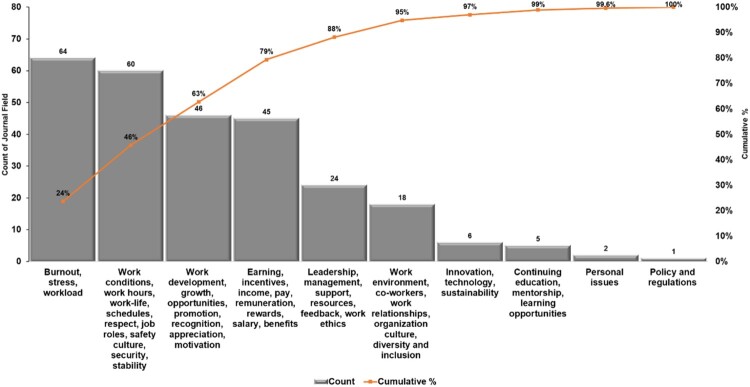
Key factors impacting pharmacy workforce satisfaction: A Pareto analysis.

Figure 6 illustrates a Pareto analysis highlighting the crucial factors impacting pharmacists’ work environments and their implications for retention and performance categorised by their frequency of mention in the included studies. Burnout, stress, and workload are the most prevalent factors, representing 24% of all occurrences, followed by work conditions and job roles at 22%. Additional significant contributors include opportunities for professional development (14%), earnings and benefits (10%), and leadership and organisational support (9%). Factors like work environment and diversity (7%), continuing education (2%), and policy-related issues (<1%) play smaller but noteworthy roles. This distribution highlights the need for targeted interventions to address high-impact factors for improving workforce satisfaction and retention.

## Discussion

The study themes highlight various factors impacting pharmacists’ satisfaction and long-term retention. Maintaining a healthy work-life balance is crucial, requiring a balance of job satisfaction, stress management, and favourable working conditions. Effective leadership and recognition play a significant role in predicting job satisfaction, emphasising the importance of supportive management and acknowledging employees’ contributions. Retention can be improved through targeted training and support programmes that promote skill development and career growth. Providing professional development opportunities, autonomy, and opportunities to apply expertise further enhances job satisfaction by empowering pharmacists. Creating a collaborative environment through addressing workplace culture, effective communication, and engagement strategies is important, as well as understanding the unique challenges within different pharmacy settings (Sallam, 2024b). Additionally, considering socio-demographic factors and generational insights provides a deeper understanding of retention trends, allowing policy reforms to address structural issues and improve satisfaction.

Regional disparities also significantly shape pharmacists’ job satisfaction, as economic conditions, healthcare policies, professional recognition, and workplace environments vary across different settings. Pharmacists in high-income countries generally report higher satisfaction due to better salaries, career advancement opportunities, and structured professional support, whereas those in low – and middle-income countries face resource limitations, heavy workloads, and inadequate financial incentives. Studies from the Middle East highlight concerns about job security and work-life balance, while Southeast Asian studies emphasise hierarchical management structures that impact workplace morale. In Africa, workforce shortages and resource constraints contribute to lower satisfaction, whereas burnout and administrative burdens are predominant concerns in North America and Europe. Public-sector pharmacists, particularly in developing regions, often express dissatisfaction due to lower salaries and limited career progression compared to private-sector counterparts, who benefit from better financial incentives and organisational support. Addressing these disparities requires region-specific workforce strategies, including policy reforms, workload management initiatives, and professional development programmes tailored to address pharmacists’ unique global challenges.

A comprehensive approach to satisfaction should include improving pay, opportunities for career advancement, and extrinsic benefits to create sustainable and fulfilling career paths for pharmacy professionals.

### Balancing job satisfaction, stress, and work conditions

Balancing job satisfaction while managing stress was a nuanced challenge faced by many pharmacy professionals. Heavy workloads, inadequate job autonomy, and persistent regulatory pressures significantly strained job satisfaction.

Lam et al. (2023) illustrated how the interplay between workload and stress led pharmacists to reconsider their career choices. High levels of psychological distress and burnout, as described by Zhao et al. (2020), were often driven by excessive job demands. Stavrou et al. (2022) and Ayele et al. (2020) found that stress and reduced job satisfaction contributed to increased turnover. However, favourable conditions such as effective leadership and work-life balance improved satisfaction, as seen in studies by Radwan et al. (2022) and Zbyrak et al. (2024). Supportive strategies like flexible work schedules reduced overtime, and better workplace culture positively impacted job satisfaction (Jegede & Ola-Olorun, 2021; Mattsson & Gustafsson, 2020). These findings highlighted the importance of strategic initiatives to ensure pharmacists felt valued and supported amidst workplace challenges.

### Leadership and recognition as predictors of job satisfaction

Having the right leadership style, such as lean or others, can offer specific advantages in enhancing employee satisfaction (Blood, 2023; Sallam, 2024a). Leadership and recognition were crucial predictors of job satisfaction within the pharmacy sector. Pharmacists responded positively to leadership characterised by support, motivation, and fairness (Azmi et al., 2019; Lama et al., 2023). Transformational leadership contributed to job satisfaction by creating an environment where pharmacists felt valued and heard (Alshammari et al., 2021; Tran et al., 2023). Recognition through appraisals, achievements, and growth opportunities also impacted satisfaction (Bondi et al., 2023; Ulutaş Deni̇z et al., 2024). Research by Mishima et al. (2022), and Dung et al. (2019) confirmed that recognition and supportive leadership enhanced job satisfaction and organisational commitment. Effective leadership that promoted collaboration and growth was essential for maintaining satisfaction and loyalty (Butt et al., 2021).

### Enhancing retention through training and support

Retention in the pharmacy field was critically tied to training opportunities and comprehensive support systems. Research consistently emphasised that structured training programmes, which included clear career ladders, personalised mentorship, and ongoing professional development, significantly bolstered retention rates among pharmacy staff. It demonstrated that implementing a career ladder and training programme substantially reduced turnover and improved employee engagement (Thames et al., 2024). These structured efforts created an environment where pharmacists felt their career was nurtured and valued. In post-pandemic contexts, Oamen (2023) highlighted that adaptive workplace practices, such as flexible hours and improved conditions, contributed to positive retention outcomes and gradual job satisfaction growth.

Furthermore, fostering environments where continuous education and skill development were prioritised supported long-term retention. Antofie et al. (2023) pointed out that supportive laws and policies promoting continuous education significantly improved job satisfaction. Citraningtyas et al. (2022) found that motivation and disciplined work practices enhanced performance and retention. In rural settings, Terry et al. (2022) identified critical factors ranging from economic support to community involvement that shaped recruitment and retention success, emphasising the importance of these strategies in under-resourced areas. Studies by Lan et al. (2019) and Smolina et al. (2021) further indicated that positive organisational climates and strong professional commitments anchored pharmacists in their roles. Asghar et al. (2024) emphasised that training significantly improved community pharmacist retention in Pakistan. Ooi et al. (2023) highlighted that job retention among community pharmacists in Malaysia was enhanced with workplace support. Wong and Kiing (2023) found that training fostered organisational commitment, aiding the retention of hospital pharmacists in Malaysia. Widhiandono et al. (2022) noted that training positively impacted job satisfaction and retention among hospital pharmacists in Indonesia. Rao et al. (2022) showed that targeted training reduced attrition among oncology pharmacists in the U.S. Collectively, these studies affirm that training and support are essential strategies for retaining pharmacists across community, hospital, and specialised sectors.

### Enhancing professional development, autonomy, and expertise application

Professional development and the opportunity to exercise autonomy were powerful drivers of job satisfaction in the pharmacy sector. Pharmacists valued roles that allowed them to apply their expertise and continue skill development. Schlosser et al. (2023), and Gulbis et al. (2024) found that autonomy, supportive schedules, and diverse professional roles correlated strongly with job satisfaction. Engagement in clinical roles and training in pharmacy informatics also enhanced job fulfilment (Abdullah Nafea Al Shammari, 2022; Rijaluddin et al., 2019). Danganan et al. (2019) showed that structured professional growth opportunities improved job satisfaction, even under moderate stress. Enhanced professional competency and purpose were reinforced by Mattsson and Gustafsson (2020), who noted that continuous professional development (CPD) was crucial for job satisfaction. Losier et al. (2021) highlighted the need to balance patient-centric engagement with adequate support to prevent burnout.

### Addressing workplace culture, communication, and engagement strategies

A positive workplace culture and effective communication were critical to pharmacists’ job satisfaction. Poor communication, exclusion, and negative workplace culture were major dissatisfaction factors (Abdullahi et al., 2023; Clabaugh et al., 2021). Promoting a culture of open communication and engagement was shown to build trust and reduce isolation (Fadare et al., 2023; Islam & Naqvi, 2023). A workplace culture that valued teamwork and included diverse perspectives improved satisfaction (Al-Omar et al., 2019; Alanazi et al., 2023). Engagement strategies that involved feedback and collaborative decision-making led to higher satisfaction and stronger workplace connections (Alomi, Bahadig, Qaism, et al., 2019). Building an inclusive culture where pharmacists felt respected and heard was essential for long-term job satisfaction and retention.

### Sector-specific job satisfaction and management preferences

Job satisfaction varied between the public and private sectors, with significant differences in compensation, job stability, and management practices. Private-sector pharmacists often experienced higher satisfaction due to better pay and supportive management (Iqbal & Iqbal, 2020; Mukosha et al., 2022). In contrast, public sector pharmacists faced challenges like limited resources, lower salaries, and fewer opportunities for career advancement (Al-Jumaili, Mohammed, et al., 2022; Berassa et al., 2021). Khaliq et al. (2021) and Maher et al. (2021) indicated that sector-specific strategies were essential to address these discrepancies and optimise job satisfaction across different work environments.

### Socio-demographic factors and generational insights for retention

Socio-demographic factors such as age, gender, and work settings influenced job satisfaction and retention among pharmacists. Different demographic groups showed varying satisfaction levels, with older pharmacists and those in academic settings often more satisfied (Alotaibi et al., 2023; Dar-Odeh et al., 2022; Renger & Niemuth, 2023). Female pharmacists frequently reported higher satisfaction than males, highlighting the impact of diverse expectations and workplace dynamics (Carvajal et al., 2019; Carvajal et al., 2021; Gu & Itoh, 2019). Younger or new pharmacists faced retention challenges due to unmet salary and career growth expectations (Ibrahim et al., 2021). Tailored strategies that considered these insights were necessary to meet the diverse needs of different demographic groups and improve retention (Iqbal & Iqbal, 2020). Interactions with supervisors, coworkers, and customers are crucial in shaping individuals’ overall job satisfaction (Horodetska et al., 2022).

### Policy reforms and structural improvements for job satisfaction

Policy reforms aimed at improving working conditions and professional structures played an essential role in boosting job satisfaction among pharmacists. Cherecheș et al. (2024), and Noha Abd Alkareem Younis (2022) emphasised the need for salary revisions, reduced workloads, and greater recognition to combat dissatisfaction. Policies that mitigated regulatory burdens were shown to create supportive environments (Ayele et al., 2020; Lynch & O'Leary, 2023). Berassa et al. (2021) indicated that incentives like salary increments and promotions led to sustained job satisfaction, particularly in the public sector. Enhanced human resources practices, highlighted by Njoku and Ogidi (2022), demonstrated that strategic changes in workplace policies could improve job satisfaction and retention. These studies collectively suggested that thoughtful structural reforms could transition job satisfaction from reactive to proactive management.

### Comprehensive factors influencing satisfaction

Understanding job satisfaction in pharmacy requires a comprehensive approach that examines factors such as work environment, interpersonal relationships, career opportunities, and stress management. Researchers like Nguyen et al. (2022) demonstrated that a blend of internal and external factors, including opportunities for growth, workload, and professional relationships, influenced job satisfaction. Ghayas et al. (2019) identified that multiple components, such as job timing, skill application, benefits, and respect for employees, contributed to overall satisfaction. This comprehensive view was supported by findings from Losier et al. (2021), who noted that pharmacists’ satisfaction varied depending on time spent on different tasks and their perceived value. Managing these factors effectively requires an integrated approach recognising the interconnectedness of workplace culture, leadership, compensation, and professional development to sustain high job satisfaction in the pharmacy field.

### Improving pay, advancement, and extrinsic benefits

Compensation and career advancement opportunities were primary motivators for job satisfaction in pharmacy roles. Shuleta-Qehaja and Kelmendi (2024) and Khaliq et al. (2021) underscored the importance of fair pay and career development. Low salaries and limited growth prospects drove dissatisfaction and turnover (Kusumah Wardani et al., 2024). Conversely, pharmacists in the private sector reported higher job satisfaction due to better compensation and career pathways (Iqbal & Iqbal, 2020; Mukosha et al., 2022). Initiatives prioritising fair pay and transparent career progression fostered a sense of security and fulfilment (Antofie et al., 2023).

### Study strengths and limitations

This systematic review has several strengths that enhance its credibility and relevance. One of its main strengths lies in its comprehensive analysis of diverse studies spanning various geographic locations, work environments, and demographic groups, providing a broad perspective on the factors influencing pharmacists’ job satisfaction and retention. Including studies using qualitative and quantitative methodologies enriched the depth of findings, allowing for a nuanced understanding of the complex interplay between job satisfaction drivers, work conditions, leadership, and professional development. Additionally, synthesising insights from research conducted pre – and post-pandemic allowed the review to capture shifts in job satisfaction and retention dynamics related to recent global disruptions, thus adding contemporary value to the discourse.

However, the review has some limitations that should be considered when interpreting the findings. One notable limitation is the variability in the methodological quality and heterogeneity of the included studies, which may have impacted the consistency and comparability of results. The reliance on published studies introduces a potential publication bias, as unpublished research and grey literature that could offer different perspectives were not included. Furthermore, while the review attempted to capture global trends, most of the literature originated from certain regions, such as North America and the Middle East, potentially limiting the applicability of findings to other areas with different healthcare systems and economic conditions. Another limitation involves the varying definitions and measurements of job satisfaction and retention across studies, which could have influenced the synthesis of results and the generalizability of conclusions. Despite these limitations, this systematic review provides valuable insights and highlights the critical need for further standardised, cross-regional research to inform policy and practice better aimed at sustaining the pharmacy workforce.

### Recommendation for future studies

Future studies should aim to adopt a more multidimensional approach when examining job satisfaction and retention among pharmacists. This includes incorporating longitudinal and cross-cultural research designs to capture the evolving nature of job satisfaction over time and across different regions (Abbas, 2024). Further investigation into the intersection of psychological well-being, work-life balance, and professional development would be valuable, especially in light of post-pandemic work adaptations. Studies should also prioritise diverse demographic groups to understand better generational and gender-based differences in satisfaction and retention (Carvajal et al., 2019). Research should examine the effects of pharmacy management practices, including technology integration and clinical pharmacy activities, on job satisfaction in various healthcare settings (Alomi, Bahadig, Aloumi, et al., 2019).

Additionally, research exploring the effectiveness of targeted workforce strategies and sector-specific interventions, including workload management, financial incentives, professional development training programmes, well-being and recognition initiatives, and policy reforms, would help solidify practical strategies to enhance workplace satisfaction and reduce turnover. Employing advanced data analytics and qualitative methods, such as in-depth interviews and focus groups, could provide richer, context-specific insights that guide meaningful changes in pharmacy practice and management. Finally, studies should explore the potential of Artificial Intelligence (AI) in optimising pharmacy work conditions, streamlining operational workflows, and supporting evidence-based management (EBMgt) strategies to enhance pharmacist satisfaction and retention (Sallam, Snygg, et al., 2024).

## Conclusion

This comprehensive review focused on synthesising findings from 81 studies to identify the predominant themes impacting job satisfaction, retention, and overall work conditions within the pharmacy sector. Studies revealed critical insights on training, leadership, workplace culture, and socio-demographic influences contributing to pharmacists’ professional fulfilment and challenges. The themes encompass the importance of enhancing professional development and supporting expertise application and the significant role of leadership, policy reforms, and sector-specific management practices.

The primary determinants of job satisfaction were intrinsic aspects of the job. People are satisfied with their work but have a high workload, inadequate salaries, and low respect. The results can significantly help pharmacy leadership, administration, and employers. Policymakers and health service managers should act to improve the quality and quantity of pharmaceutical care services. Developing and implementing a well-framed system that provides a conducive working environment, remuneration, and greater autonomy could improve job and career satisfaction.

Addressing job satisfaction offers a deeper understanding of the complex dynamics pharmacists face in diverse work settings, guiding targeted strategies for sustainable improvement in job retention and satisfaction.

## Data Availability

Data is available upon valid request.
